# Enhanced Adsorption of Xylenol Orange by CTAB-Functionalized Tomato-Derived Biochar: Mechanisms and Performance Evaluation

**DOI:** 10.3390/molecules31040708

**Published:** 2026-02-18

**Authors:** Shirui Zheng, Mengjie Bai, Yongwei Li, Runxiu He, Wenxu Wang, Yanping Feng, Haijie Wang, Fangfang Liu, Zhihao Fang

**Affiliations:** 1 State Key Laboratory of Bio-Fibers and Eco-Textiles, College of Materials Science and Engineering, Shandong Collaborative Innovation Center of Marine Biobased Fibers and Ecological Textiles, Qingdao University, Qingdao 266071, China; threezheng@aliyun.com; 2 School of Chemical Engineering and Environment, Weifang University of Science and Technology, Weifang 262700, China; 13406048016@163.com (M.B.); 18751180307@163.com (R.H.); wangwenxu201@gmail.com (W.W.); 13721985173@163.com (Y.F.); wanghaijie1989122@163.com (H.W.); liuff10507@wfust.edu.cn (F.L.); 3 Shandong Solid New Material Technology Co., Ltd., Weifang 262700, China

**Keywords:** tomato biochar, CTAB@TBC, dye, wastewater treatment, agricultural residue

## Abstract

In this work, a tomato biochar composite modified with cetyltrimethylammonium bromide (CTAB@TBC) was successfully synthesized using an ethanol solution method. It was comprehensively characterized to evaluate its morphology (SEM), crystal structure (XRD), chemical bonding (FT-IR and Raman spectroscopy), surface area (BET analysis), and thermal stability (TGA). The adsorption performance of the composite for xylenol orange (XO) was subsequently evaluated in detail. The findings revealed that CTAB@TBC exhibited a maximum adsorption capacity of 150.59 mg/g for XO. Kinetic analysis indicated that the adsorption process conformed to a pseudo-second-order model, implying that chemisorption was the step limiting the rate. The Langmuir model accurately described the adsorption isotherm data, suggesting monolayer adsorption on a uniform surface. Thermodynamic evaluation further revealed a negative Gibbs free energy (ΔG) change, confirming the spontaneity of the adsorption process. In summary, the results suggest that CTAB@TBC is a highly effective adsorbent for eliminating dyes from wastewater and offers considerable promise for treating industrial effluents.

## 1. Introduction

Rapid industrialization and population growth have rendered dye wastewater pollution a critical global environmental challenge [1]. Synthetic dyes are extensively utilized in various industries, such as textiles, printing, pharmaceuticals, paper, plastics, and cosmetics [2]. Their discharge—frequently without adequate treatment—poses severe threats to aquatic ecosystems and human health [3,4]. These effluents, containing complex organic dyes, exhibit high toxicity, structural stability, persistence, and resistance to biodegradation [5,6,7]. Consequently, they cause esthetic degradation [8], reduce light penetration [9], inhibit aquatic photosynthesis, promote eutrophication and mutagenic effects (even at low concentrations) [10], and endanger human health through the food chain [11]. In this context, the development of highly efficient, economically feasible, and environmentally friendly treatment technologies has become essential.

Among various treatment methods, adsorption has gained significant attention due to its simplicity, high efficiency, recyclability, and cost-effectiveness [12,13,14]. Against this backdrop, biochar—a carbon-dense substance produced via thermochemical conversion (e.g., pyrolysis) of biomass—has proven to be a viable adsorbent for dye wastewater remediation. Its advantages include abundant and low-cost raw material sources (e.g., agricultural waste), inherent porous structure, large specific surface area, and environmental friendliness [15,16,17], making it an attractive alternative to conventional adsorbents. However, pristine biochar often exhibits limited adsorption capacity for anionic dyes [18,19], primarily due to its predominantly negative surface charge (leading to electrostatic repulsion) and lack of specific functional groups. This inherent limitation restricts its widespread practical application, necessitating surface modification to enhance performance.

To address these limitations, surface modification of biochar has been widely explored [20,21,22], with cationic surfactants like cetyltrimethylammonium bromide (CTAB) proving particularly effective. CTAB—a quaternary ammonium compound—adsorbs onto the biochar surface via electrostatic interactions and hydrophobic bonding, forming monolayer or bilayer structures. This modification not only reverses the biochar’s surface charge from negative to positive (strengthening electrostatic attraction to anionic dyes) but also enhances hydrophobicity, increases specific surface area, and introduces new adsorption sites [23,24]. As a result, CTAB-modified biochar demonstrates superior adsorption capacity, selectivity, and reusability for anionic dyes, broadening its applicability in complex wastewater treatment.

XO, a representative triphenylmethane anionic dye, was selected to evaluate the adsorption performance of CTAB-modified biochar [25]. Owing to its widespread applications in textile dyeing, in paper manufacturing, and as a complexometric indicator in analytical chemistry, the discharge of XO into wastewater streams poses significant environmental and health concerns [26,27]. Its complex molecular structure and anionic nature make it a challenging pollutant to remove, thus serving as a representative target for assessing the efficacy of modified adsorbents in treating complex dye effluents [28,29]. This investigation aims to provide valuable insights for the development of efficient and sustainable adsorbents for the treatment of triphenylmethane anionic dye wastewater.

In this study, tomato-derived biochar (TBC) was produced through pyrolysis at 400 °C under a nitrogen atmosphere. Subsequently, CTAB@TBC was prepared by modifying TBC with a CTAB ethanol solution. In comparison to traditional aqueous-phase intercalation, this approach enhances CTAB permeability and dispersibility within biochar pores, prevents agglomeration, preserves the porous structure of biochar, and improves the utilization efficiency of adsorption sites. The composite material was then employed to remove XO from simulated wastewater. The adsorption performance and underlying mechanisms under various conditions were systematically investigated through a series of experiments with complementary characterization techniques. The adsorption kinetics, isotherm characteristics, and thermodynamic properties of XO on CTAB@TBC were thoroughly investigated. Furthermore, the regeneration capability and reusability of CTAB@TBC were evaluated to assess its practical applicability. This study presents a viable strategy for utilizing agricultural waste by converting vegetable-based straw into an efficient and recyclable adsorbent, thereby contributing to resource utilization and environmental pollution mitigation.

## 2. Results and Discussion

### 2.1. Characterization of the Adsorbent

**Figure 1:**
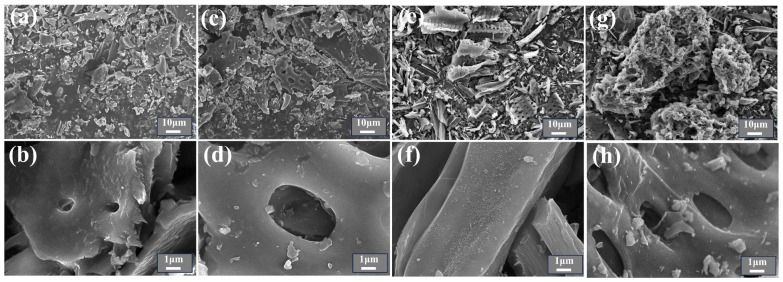
(**a**,**b**) SEM image of TBC, (**c**,**d**) SEM image of CTAB_0.1_@TBC, (**e**,**f**) SEM image of CTAB_0.3_@TBC, (**g**,**h**) SEM image of CTAB_0.5_@TBC.

The SEM images of TBC and CTAB@TBC are presented in Figure 1. As shown in Figure 1a–h, TBC exhibits a well-defined, orderly structure with discernible regular pore arrangements. In contrast, CTAB@TBC displays a looser and more porous morphology, characterized by increased structural complexity and irregular pore distribution. CTAB modification significantly enhances the biochar’s surface roughness, transforming the originally smooth surface into one covered with numerous fine granular particles. This observation confirms that CTAB has been effectively loaded onto the biochar surface [9]. As the CTAB content increases, the surface roughness of TBC increases, and CTAB aggregation becomes more pronounced. The morphological transformation, marked by particle deposition and increased roughness, may create additional active or adsorption sites, thereby improving the material’s adsorption performance and related properties.

**Figure 2:**
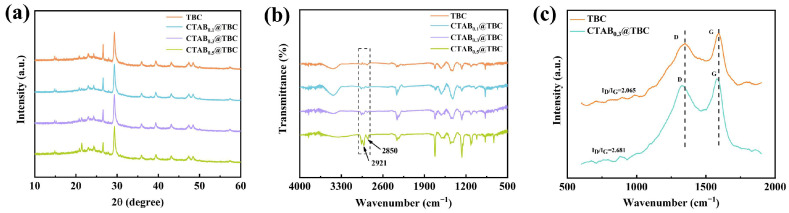
(**a**) XRD spectra of TBC and CTAB_x_@TBC composites, (**b**) FTIR patterns of TBC and CTAB_x_@TBC composites, (**c**) Raman spectroscopy of TBC and CTAB_0.3_@TBC.

The XRD patterns of TBC and CTAB@TBC are illustrated in Figure 2a. All samples exhibit nearly identical diffraction patterns, indicating that the CTAB surface modification does not affect the crystalline structure of TBC. The XRD data reveal diffraction peaks that suggest the presence of amorphous carbon in TBC. A broad peak observed at approximately 23.4° is attributed to amorphous carbon, while the weak diffraction peaks observed at 20.7°, 22.9°, and 43.2° correspond to the characteristic diffraction of the graphite-like carbon’s microcrystalline structure. The diffraction peaks at 29.3°, 35.9°, 39.3°, 43.1°, 47.3°, and 48.4° correspond to the standard characteristic peaks of calcite-type calcium carbonate (CaCO_3_), confirming the presence of calcium carbonate in the sample [5,22].

Figure 2b shows the FTIR spectra of TBC and CTAB@TBC. Comparative analysis of their spectral features indicates that both samples exhibit distinct vibrational absorption bands. Notably, the FTIR spectrum of CTAB@TBC displays two additional characteristic peaks at 2850 cm^−1^ and 2921 cm^−1^, which are absent in the TBC spectrum. These peaks correspond to the symmetric and asymmetric stretching vibrations of methylene (–CH_2_–) groups, respectively, arising from CTAB molecules immobilized on the TBC surface [12,14], the intensity of the –CH_2_– stretching vibration peak increases significantly with increasing CTAB proportion. This observation provides clear spectroscopic evidence for the successful incorporation of CTAB into the TBC matrix.

Figure 2c displays the Raman spectra of TBC and CTAB_0.3_@TBC, featuring characteristic D (1345 cm^−1^) and G (1593 cm^−1^) bands. The D band is linked to structural disorder and localized defects in graphite, originating from the vibrational modes of sp^3^-hybridized carbon atoms. In contrast, the G band corresponds to the stretching vibrations of sp^2^-hybridized carbon atoms within graphene’s two-dimensional hexagonal lattice [30]. The intensity ratio of the D band to the G band (ID/IG) indicates the relative levels of structural defects and graphitic ordering in biochar. The ID/IG values for TBC and CTAB_0.3_@TBC were 2.065 and 2.681, respectively, demonstrating increased structural disorder in CTAB_0.3_@TBC that facilitates the adsorption process. CTAB_0.3_@TBC exhibits the highest degree of structural disorder among the materials studied.

**Figure 3:**
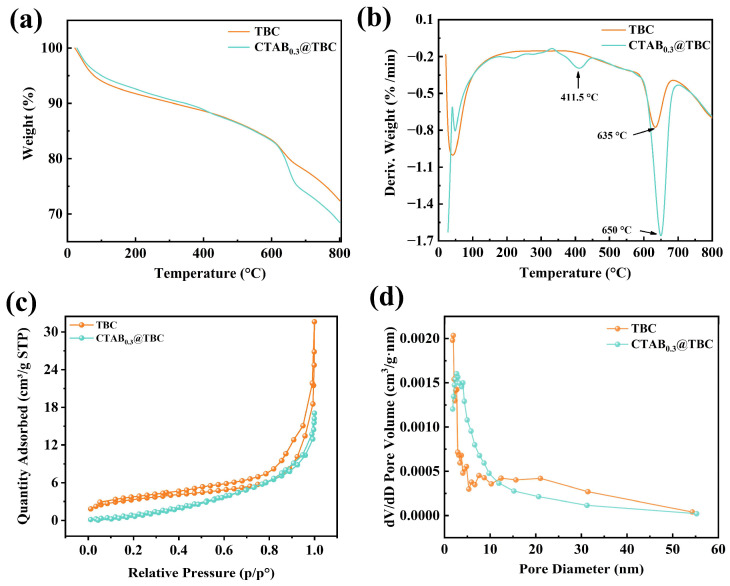
(**a**) TG of TBC and CTAB_0.3_@TBC, (**b**) DTG of TBC and CTAB_0.3_@TBC, (**c**) N_2_ adsorption–desorption isotherms of TBC and CTAB_0.3_@TBC, (**d**) pore size distributions of TBC and CTAB_0.3_@TBC.

The TGA and DTG curves of TBC and CTAB_0.3_@TBC are presented in Figure 3a,b. For TBC, an initial mass loss of 5.94% at sub-10 °C temperatures is attributed to the vaporization of physically adsorbed moisture. A slow mass reduction of 12.81% between 100 °C and 630 °C is attributed to the decomposition of organic components such as hemicellulose, cellulose, and lignin remaining in the biochar. Above 630 °C, an 8.87% mass loss indicates the slow reformation and graphitization of highly stable aromatic carbon structures, as well as the decomposition of a small amount of inorganic carbonates [31]. The thermal decomposition trends of CTAB_0.3_@TBC and TBC are similar. Below 100 °C, a mass loss of 4.98% occurs due to the volatilization of physically adsorbed water. Between 100 °C and 650 °C, the mass loss is 16.74%, primarily caused by the thermal decomposition of CTAB and organic substances such as cellulose and lignin remaining in the biochar. Above 650 °C, a mass loss of approximately 9.80% suggests the gradual decomposition and structural reorganization of the highly stable aromatic carbon framework. Additionally, A characteristic peak at 411.5 °C in the DTG profile of CTAB_0.3_@TBC is attributed to the thermal degradation of CTAB [30].

Figure 3c,d present the N_2_ adsorption–desorption isotherms and pore size distribution profiles of TBC and CTAB_0.3_@TBC. Both TBC and CTAB_0.3_@TBC exhibit mesoporous structures (2–50 nm) featuring slit-shaped or irregularly stacked pores, as evidenced by Type IV adsorption isotherms with H3 hysteresis loops. Quantitative analysis reveals that the specific surface area of the CTAB-modified TBC decreased from 57.39 m^2^/g to 21.93 m^2^/g, whereas the average pore diameter increased from 17.27 nm to 43.62 nm. This change is primarily attributed to the adsorption of CTAB, a long-chain cationic surfactant, onto the biochar surface through electrostatic interactions, hydrophobic effects, and van der Waals forces. During this process, CTAB molecules also enter certain micropores and mesopores, partially blocking the pore channels and thereby reducing the accessible surface area. CTAB modification led to an overall reduction in the N_2_ adsorption capacity of CTAB_0.3_@TBC, particularly in the high P/P_0_ region. This phenomenon indicates a decrease in total pore volume, which is attributed to the partial pore blockage caused by the intrusion of CTAB molecules into the pore structure. These observations confirm the successful modification of the biochar by CTAB [22].

### 2.2. Adsorption Experiment

#### 2.2.1. Performance Screening of Different Adsorption Materials

**Figure 4:**
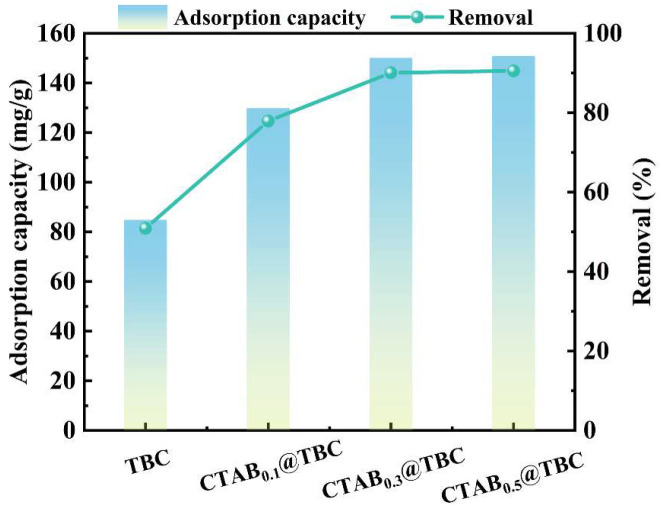
Adsorbent screening for XO removal.

The adsorption performance of the synthesized material CTAB@TBC toward XO was evaluated by comparative experiments, as shown in Figure 4. Pristine TBC exhibited a removal efficiency of 50.9% and an adsorption capacity of 84.83 mg/g. As the CTAB content increased, both the removal efficiency and adsorption capacity of CTAB@TBC were enhanced. Specifically, the removal efficiency increased from 77.90% for CTAB_0.1_@TBC to 90.07% for CTAB_0.3_@TBC, while the adsorption capacity rose from 129.83 to 150.11 mg/g. However, further increases in CTAB content did not lead to additional improvement, and CTAB_0.5_@TBC showed no significant advantage over CTAB_0.3_@TBC. These results indicate that an appropriate CTAB loading can significantly enhance the adsorption performance of TBC. Therefore, CTAB_0.3_@TBC was selected for subsequent in-depth studies.

#### 2.2.2. Impact of Initial pH on Adsorption

**Figure 5:**
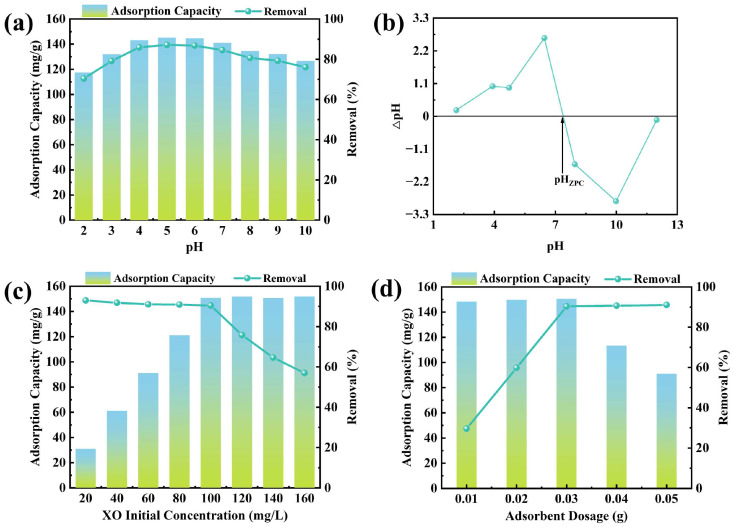
(**a**) Impact of pH on adsorption, (**b**) pH_ZPC_ measurement for CTAB_0.3_@TBC, (**c**) impact of adsorbent dose on adsorption, and (**d**) impact of initial XO concentration on adsorption.

The impact of the solution’s initial pH on XO adsorption by CTAB@TBC was evaluated (Figure 5a). Adsorption capacity and removal efficiency exhibited clear pH dependence: both increased across pH 2.0–5.0 and then declined progressively over the range 5.0–10.0. This phenomenon arises from the combined influence of the surface charge of CTAB@TBC and the pH-dependent dissociation behavior of XO. As the pH changes in aqueous solution, XO transitions among various ionic species (e.g., H_4_XO^+^, H_4_XO, H_3_XO^−^, HXO^3−^, XO^4−^). These variations indicate that adsorption is governed not only by electrostatic interactions but also by potential chemical interactions. Overall, CTAB@TBC exhibits an effective pH range of 4.0 to 6.0 for XO adsorption. The adsorption mechanism can be clarified by examining the zero-point charge (ZPC) of CTAB@TBC, which was determined to be 7.39 (Figure 5b). When the solution pH is lower than this value, the CTAB@TBC surface becomes positively charged, which favors the adsorption of anionic XO. Conversely, at pH values above pH_ZPC_, the surface becomes negatively charged and the electrostatic attraction between the surface and XO is consequently weakened, leading to reduced adsorption [1]. Based on the above analysis, the pH value of the adsorption solution was adjusted to 6.0 to ensure the best adsorption effect.

#### 2.2.3. Impact of CTAB@TBC Dosage on Adsorption

The optimal CTAB@TBC dosage was determined by evaluating how different application levels influenced the adsorption capacity and removal efficiency of XO. As depicted in Figure 5c, raising the CTAB@TBC dosage from 0.01 g to 0.03 g markedly improved the removal efficiency of XO, increasing it from 29.67% to 90.32%. Beyond 0.03 g, however, the efficiency remained essentially unchanged, indicating a saturation point. When the dosage was increased from 0.03 g to 0.05 g, the adsorption capacity decreased significantly, from 150.54 mg/g to 91.05 mg/g. This decrease can be attributed to the limited availability of dye molecules at higher adsorbent concentrations, which prevents the full utilization of adsorption sites [22]. Therefore, when adsorption equilibrium is reached at the solid–liquid interface, the adsorption capacity per unit mass of the adsorbent decreases.

#### 2.2.4. Impact of XO’s Initial Concentration on Adsorption

The initial concentration of XO within the reaction system plays a critical role in determining the adsorption performance of the synthesized adsorbent. As shown in Figure 5d, varying the starting XO concentration markedly influences the adsorption behavior of CTAB@TBC. As the initial concentration of XO increased from 20 to 160 mg/L, the equilibrium adsorption capacity increased from 30.99 to 150.80 mg/g and then approached a plateau. This behavior is attributed to the progressive saturation of active sites on the adsorbent surface. At higher XO concentrations, the larger concentration gradient provides a stronger driving force for mass transfer, thereby accelerating the diffusion of XO molecules toward the adsorbent surface [12,19]. However, when the initial XO concentration exceeds 100 mg/L, the dye removal efficiency significantly decreases. Based on this trend, 100 mg/L can be identified as the optimal initial XO concentration for achieving maximum adsorption performance.

#### 2.2.5. Impact of Co-Existing Ions on Adsorption Process

**Figure 6:**
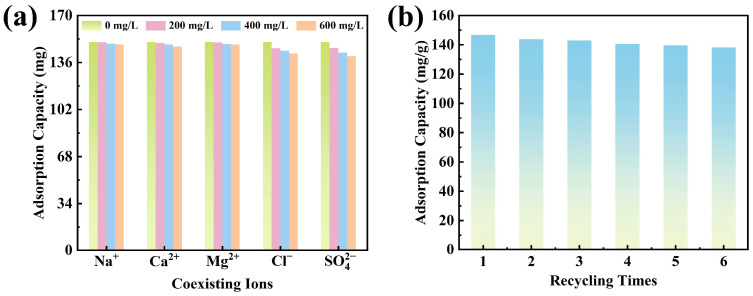
(**a**) Impact of co-existing ions on adsorption process by CTAB@TBC. (**b**) Impact of CTAB@TBC regeneration on its adsorption capacity for XO.

To evaluate the stability and anti-interference performance of CTAB@TBC toward XO adsorption under practical application conditions, a competitive ion coexistence experiment was conducted. As illustrated in Figure 6a, elevating the levels of coexisting cations produced only a minimal influence on the adsorption of XO by CTAB@TBC. This minimal impact is attributed to the presence of metal ions in aqueous solution primarily as positively charged species or hydrated M(OH)_x_ complexes, which cannot effectively compete with anionic dye molecules for the available adsorption sites on CTAB@TBC [32]. In contrast, the presence of anions reduced XO uptake by vying for the positively charged sites available on CTAB@TBC, which also weakened the electrostatic forces responsible for adsorption. Experimental findings revealed a competitive sequence of Cl^−^ < SO_4_^2−^. The monovalent chloride ion, having relatively low charge density, shows weak competitiveness, whereas sulfate, a divalent anion with a high charge density, which results in stronger electrostatic attraction. Consequently, its inhibitory effect on related processes is also more pronounced [22].

#### 2.2.6. Reusability of CTAB@TBC

Evaluating the cyclic stability of the CTAB@TBC adsorbent during XO removal is crucial for assessing its practical applicability and long-term effectiveness. For regeneration, the adsorbent was desorbed with 0.1 mol/L NaOH solution after each adsorption experiment. This adsorption–desorption cycle was repeated six times, and the resulting recyclability performance is presented in Figure 6b.

Across successive adsorption–desorption cycles, CTAB@TBC showed a slight reduction in its adsorption capacity for XO. After six adsorption–desorption cycles, the adsorption capacity of CTAB@TBC decreased from 150.82 to 138.12 mg/g, retaining 91.58% of its initial value. This reduction in adsorption efficiency can be attributed to the partial loss of CTAB from the interlayer structure of TBC during desorption, which decreases the number of available active sites. Nevertheless, CTAB@TBC maintained a high adsorption capacity after six cycles, demonstrating good regenerability and promising potential for reuse in practical applications.

To validate the practical applicability of CTAB@TBC, its technical and economic performance was benchmarked against commercial sorbents (e.g., activated carbon, ion-exchange resins) widely used for XO removal. Technically, CTAB@TBC exhibits a superior adsorption capacity (150.82 mg/g) and maintains exceptional cyclic stability, retaining 91.58% of its initial capacity after six cycles. Economically, CTAB@TBC offers a competitive advantage owing to low-cost precursors (TBC and CTAB) and a straightforward synthesis protocol, collectively reducing production costs compared to advanced functional sorbents. In contrast, activated carbon requires energy-intensive activation and regeneration, while ion-exchange resins entail higher synthesis expenses and inferior cyclic stability for anionic dyes such as XO.

After CTAB-modified biochar reaches adsorption saturation, incineration treatment can facilitate the complete mineralization of adsorbed organic dyes, thus effectively eliminating the risk of secondary pollution. Meanwhile, this process enables the recovery of valuable components from incineration ash and the energy utilization of high-carbon substrates, thereby constructing an integrated closed-loop process of “adsorption-incineration-resource recovery”. This study provides a sustainable strategy for dye wastewater treatment and the high-value utilization of biomass resources.

### 2.3. Adsorption Kinetics

A comprehensive understanding of adsorption kinetics is essential for elucidating the mechanisms governing the interactions between XO molecules and the CTAB@TBC surface. To systematically investigate the time-dependent behavior of these interactions, batch adsorption experiments were conducted at a constant temperature of 25 °C with an initial XO concentration of 100 mg/L. The kinetic data were analyzed using pseudo–first-order and pseudo–second-order models, as well as an intraparticle diffusion model, to elucidate the mechanisms that control the adsorption rate. To explore the kinetic mechanism of the adsorption process, this study employed the pseudo-first-order kinetic model (Equation (1)) and pseudo-second-order kinetic model (Equation (2)), respectively, to characterize the potentially involved physical and chemical adsorption behaviors. In addition, to further elucidate the mass transfer characteristics of XO molecules within the CTAB@TBC material, the intra-particle diffusion model (Equation (3)) was employed to analyze the internal diffusion and any concomitant proton transfer. Subsequently, the experimental data were fitted nonlinearly using the kinetic Equations (1)–(3) [19,20,22].

(1) $$q_{t}={\;q}_{e}(1-e^{{-k}_{1}t})$$

(2) $$q_{t}=\frac{q_{e}^{2}k_{2}t}{1+q_{e}k_{2}t}$$

(3) $$q_{t}=k_{i}t^{0.5}+C$$

where q_t_ and q_e_ represent the adsorption capacity at contact time t and at adsorption equilibrium, respectively (mg/g). k_1_, k_2_ and k_i_ refer to the rate constants of the pseudo-first-order kinetic model (1/min), the pseudo-second-order kinetic model (1/min) and the intraparticle diffusion adsorption model (mg/g·min0.5), respectively, t refers to the adsorption time in the adsorption process (min), C is the boundary layer effect constant in the intraparticle diffusion model (mg/g).

**Figure 7:**
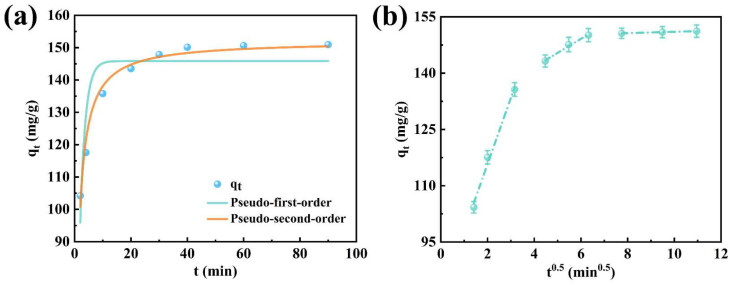
(**a**) Model fitting of XO adsorption on CTAB@TBC using the pseudo-first-order and pseudo-second-order kinetic equations; (**b**) evaluation of XO adsorption on CTAB@TBC through the intraparticle diffusion model.

As illustrated in Figure 7, the pseudo-first-order kinetic, pseudo-second-order kinetic, and intra-particle diffusion models were used to analyze the kinetic behavior of the adsorption process. The rate constants k_1_, k_2_, and k_i_ for each model were determined through linear regression. The relevant parameters were calculated according to Equations (3)–(5) and summarized in Table 1. The analysis of goodness of fit (R^2^) and the evaluation results of deviations between the calculated and measured values indicated that the pseudo-second-order kinetic model had a significantly better ability to describe the experimental data than the pseudo-first-order model, suggesting that the adsorption process was more consistent with the pseudo-second-order reaction mechanism. Furthermore, the kinetic parameters suggest that XO adsorption onto CTAB@TBC adheres to a pseudo-second-order kinetic model. The close correspondence between the calculated and experimental values indicates that chemisorption is likely the primary rate-controlling step, involving valence-based interactions through electron sharing or exchange between the CTAB-modified TBC surface and XO molecules [20,22].

Figure 7b and Table 1 display the fitted curves and corresponding parameters of the intraparticle diffusion model, offering insight into contaminant-transport behavior and enabling identification of the rate-controlling steps in the adsorption process. The adsorption process can be divided into three sequential phases: Phase I (0–10 min) is characterized by a fast adsorption step mainly controlled by film-layer diffusion, during which plentiful reactive groups (such as amino groups) together with the porous framework of CTAB@TBC promote effective XO adsorption through electrostatic attraction. Phase II (10–40 min) represents the period in which intraparticle diffusion becomes the predominant mechanism, during which XO gradually moves into the mesoporous framework of CTAB@TBC. The modest decline in diffusion rate indicates that adsorption continues, supported by the adsorbent’s internal pore structure. Phase III (>60 min) marks the onset of adsorption equilibrium, during which the uptake rate declines sharply as the internal active sites become nearly saturated. The considerable intercept (C) observed in the diffusion graph indicates strong boundary-layer resistance and restricted pore accessibility, both of which hinder additional adsorption. Overall, these findings show that CTAB@TBC makes full use of its accessible surface area and pore structure during the adsorption process [19].

When intraparticle diffusion becomes the rate-limiting step, the overall adsorption rate is primarily governed by the mass transfer of pollutants within the composite porous network structure of the CTAB@TBC adsorbent. The experimental data exhibit a strong fit with the intraparticle diffusion model, suggesting that this mass transfer mechanism plays a dominant role in regulating the adsorption performance of CTAB@TBC. Further analysis, combining the pseudo-second-order kinetic model and the intraparticle diffusion model, reveals that the adsorption behavior of XO onto CTAB@TBC follows a dual mechanism involving both chemisorption and diffusion control. Specifically, chemisorption is represented by the chemical interactions between the adsorbate and the surface functional groups of the adsorbent, while diffusion control reflects the mass transfer resistance encountered by the adsorbate molecules as they migrate through the porous medium. The synergistic effect of the above mechanisms not only endows CTAB@TBC with excellent adsorption capacity, especially high removal efficiency under low-concentration pollutant conditions, but also highlights its potential for practical water treatment applications [19].

### 2.4. Adsorption Isotherms

The adsorption isotherm describes the relationship between the equilibrium concentration of XO in solution and the amount adsorbed by CTAB@TBC at a given temperature. The analysis of this isotherm is essential for elucidating the partitioning behavior of dye molecules between the liquid phase and the adsorbent, and it provides a deeper understanding of the adsorption mechanism, surface properties, and binding strength of modified TBC. To gain a deeper understanding of how XO is removed, the interactions between the modified TBC and the solution were further analyzed through isotherm modeling. Batch adsorption experiments were conducted at pH 6 with varying initial concentrations of XO. To evaluate the adsorption data, the Langmuir and Freundlich isotherm models were employed, each offering complementary insight into the fundamental adsorption behavior. The Langmuir model (Equation (4)) assumes that adsorption occurs as a single molecular layer on a homogeneous surface, while the Freundlich model (Equation (5)) serves as an empirical description of adsorption taking place on non-uniform surfaces with reversible binding of molecules [3,14].

(4) $$\mathrm{Langmuir}\;\mathrm{model}:\;q_{e}=\frac{QK_{L}C_{e}}{1+{\;K}_{L}C_{e}}$$

(5) $$\mathrm{Freundlich}\;\mathrm{model}:\;q_{e}=K_{F}C_{e}^{\frac{1}{n}}$$

where K_L_ denotes the Langmuir constant related to adsorption property (L/mg), K_F_ represents Freundlich constant indicative of adsorption capacity (mg/g), q_e_ is the adsorption capacity at equilibrium (mg/g), and C_e_ denotes the equilibrium concentration of XO that persists in the solution (mg/L).

**Figure 8:**
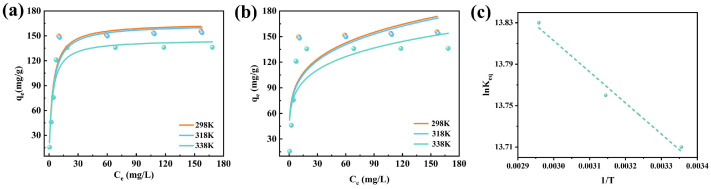
Adsorption isotherm fit of XO adsorption onto CTAB@TBC (**a**) Langmuir and (**b**) Freundlich. (**c**) Van’t Hoff Curve of CTAB@TBC for XO adsorption.

Figure 8 presents the fitting results of the adsorption behavior of XO on CTAB@TBC according to the Langmuir and Freundlich isotherm models. The corresponding model parameters and coefficients of determination (R^2^) are provided in Table 2. The Langmuir model fitted the experimental results better than the Freundlich model, indicating the uniform adsorption of XO dye molecules on the active sites of CTAB@TBC [14], which implies the formation of monolayer adsorption with a limited capacity due to the finite active sites. A relatively large K_L_ value (in the unified measurement unit system) reflects a high affinity between CTAB@TBC and XO, endowing the adsorbent with a high adsorption capacity even at low XO concentrations. This type of adsorption is typically irreversible and forms a single molecular layer. The good fitting of experimental data to the Langmuir model verifies this monolayer adsorption mechanism, demonstrating that CTAB@TBC has uniformly distributed surface active sites that only allow monolayer adsorption of XO molecules [3].

### 2.5. Thermodynamics of Adsorption

To investigate the thermodynamic mechanism underlying the adsorption process, this study applied the Langmuir model, which provides the best fit for the adsorption isotherm of XO onto CTAB@TBC, to determine the relevant thermodynamic parameters, including the Gibbs free energy change (ΔG), enthalpy change (ΔH), and entropy change (ΔS). These values were calculated using Equations (6)–(8), following the procedures outlined in previous studies [12,33].

(6) $$∆G=-RT\ln K_{eq}$$

(7) $$\ln K_{eq}=\frac{∆S}{R}-\frac{∆H}{RT}$$

(8) $$K_{eq}=K_{L}M_{XO}$$

where K_eq_ denotes the equilibrium constant for adsorption, R refers to the universal gas constant (8.314 J/mol·K), T indicates for the absolute temperature in Kelvin, M_XO_ is the molar weight of XO (mg/mol).

The adsorption thermodynamic curve, presented in Figure 8c, demonstrates that the adsorption behavior follows the Van’t Hoff model. Based on the fitted line, the ΔH and ΔS were derived from its slope and intercept. A complete set of thermodynamic parameters is provided in Table 3. The positive ΔH indicates that the adsorption of XO onto CTAB@TBC is an endothermic process, while the negative ΔG confirms that the adsorption proceeds spontaneously under the experimental conditions. In addition, the further decrease in ΔG with increasing temperature suggests that higher temperatures enhance the spontaneity of the adsorption process [19]. The positive value of ΔS indicates that the solid–liquid interface becomes more disordered as XO is adsorbed [12]. Taken together, these findings demonstrate that the adsorption of XO on CTAB@TBC occurs spontaneously, driven by combined enthalpic and entropic contributions that affirm its thermodynamic favorability under the examined conditions.

### 2.6. Adsorption Mechanism of XO on CTAB@TBC

Based on the analyses of adsorption kinetics (Section 2.3) and adsorption isotherms (Section 2.4), the adsorption of XO onto CTAB@TBC primarily follows a monolayer physicochemical process dominated by chemical adsorption. This process also involves physical mechanisms, including surface adsorption, intraparticle diffusion, and electrostatic interactions. The numerous active sites on the CTAB@TBC surface promote the adsorption of XO molecules, which mainly arises from van der Waals and other intermolecular forces between the material surface and XO molecules. TBC possesses a well-developed mesoporous structure (average pore diameter 29.60 nm), which is substantially larger than the Stokes radius (0.129 nm) and the hydrated radius (0.335 nm) of XO, allowing XO molecules to freely access the pores [34,35]. CTAB modification imparts a positive surface charge to TBC, facilitating efficient adsorption of anionic XO via electrostatic attraction. Furthermore, lower temperatures favor physical adsorption. The synergistic effect of these mechanisms enables CTAB@TBC to retain high adsorption performance at low temperatures.

Then, the adsorption mechanism was investigated by comparing the FT-IR and XPS characteristics before and after XO adsorption, with a systematic assessment of the contributions of physical adsorption, electrostatic attraction, hydrophobic interaction, and complexation.

**Figure 9:**
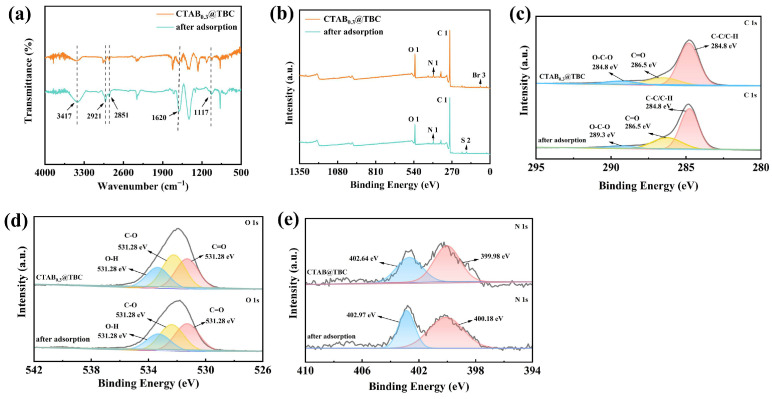
(**a**) FTIR patterns of CTAB_0.3_@TBC and CTAB_0.3_@TBC with XO adsorbed; (**b**–**e**) the XPS spectra of CTAB_0.3_@TBC before and after XO adsorption.

As shown in Figure 9a, the broad O–H stretching vibration peak at 3417 cm^−1^ is attributed to the –OH or –SO_3_^−^ groups of XO interacting with hydrogen atoms on the surface of CTAB@TBC via hydrogen bonding, which represents a typical physical adsorption interaction. Additionally, the decreased intensity of the C–H stretching vibration peaks of CTAB (2919.6 and 2850.2 cm^−1^) suggests a conformational change in the alkyl chain induced by XO adsorption, reflecting hydrophobic interactions between the alkyl tails of CTAB and the aromatic rings of XO. The peak at 1620 cm^−1^ corresponds to π–π stacking between the C=C bonds in the XO benzene ring and the aromatic ring of biochar, another physical adsorption force that enhances the binding affinity. The peak at 1116.5 cm^−1^ is associated with the S=O stretching vibration of the sulfonic acid group in XO, confirming the presence of XO on the material surface [12,36]. These FT-IR results collectively indicate that both physical adsorption (hydrogen bonding, π–π stacking) and hydrophobic interactions contribute to the initial adsorption stage.

The XPS spectra of CTAB@TBC and CTAB@TBC with XO adsorbed (Figure 9b–e) further reveal the multi-stage nature of the adsorption process. As shown in Figure 9b, the appearance of a new S 2p characteristic peak (corresponding to the SO_3_^−^ group in XO) and the disappearance of the Br 3d peak (corresponding to Br^−^ in CTAB) confirm an ion exchange process, which is a form of electrostatic attraction. This ion exchange represents a dominant chemical interaction in the intermediate stage of adsorption. The C 1s spectrum exhibited no significant changes, indicating that the carbon backbone did not participate in the adsorption process, which supports that the main interactions occur at the functional groups rather than the carbon skeleton [36]. In the O 1s spectrum, the increased proportion of the C=O component (from 35.47% to 38.88%) after XO adsorption suggests the formation of coordinate bonds between the carboxyl groups of XO and the surface sites of CTAB@TBC, indicating a contribution from complexation. The decreased proportion of C–O (from 39.88% to 37.34%) reflects the consumption of surface hydroxyl groups to form hydrogen bonds with XO, further verifying the role of physical adsorption. The slight decrease in adsorbed water (from 24.65% to 23.78%) implies dehydration during adsorption, which is consistent with the strengthening of surface interactions [12]. In the N 1s spectrum, the positive shift in binding energies of –NH_2_ (from 399.98 eV to 400.18 eV) and –NH_3_^+^ (from 402.64 eV to 402.97 eV) indicates a reduction in electron cloud density around nitrogen atoms, which is attributed to electrostatic attraction between the quaternary ammonium groups of CTAB and the sulfonic acid groups of XO [35,36]. This electrostatic interaction is a dominant force in the later stage of adsorption, stabilizing the adsorbed XO molecules.

Overall, the adsorption process of XO onto CTAB@TBC involves multiple synergistic mechanisms: In the initial stage, physical adsorption (hydrogen bonding, π–π stacking) and hydrophobic interactions dominate, driving the rapid attachment of XO to the material surface. In the intermediate stage, ion exchange (a form of electrostatic attraction) becomes prominent, facilitating the replacement of Br^−^ by SO_3_^−^ and enhancing the binding strength. In the final stage, electrostatic attraction and complexation stabilize the adsorbed XO, with the quaternary ammonium groups and surface carboxyl groups playing key roles.

**Figure 10:**
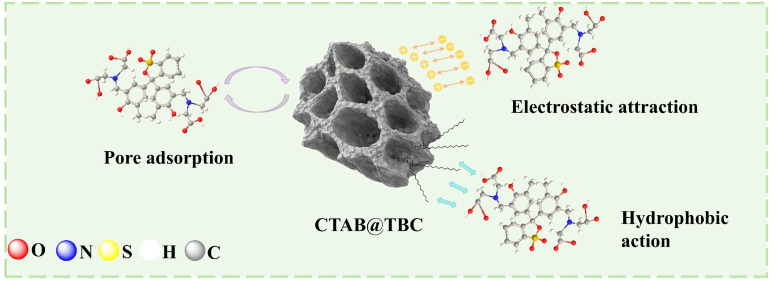
Mechanism of XO adsorption on CTAB@TBC.

Based on a comprehensive assessment of the characterization results and experimental data, we put forward a reasonable explanation of how XO adsorbs onto CTAB@TBC, as depicted in Figure 10.

### 2.7. Comparative Assessment of XO Removal Performance Across Diverse Adsorbents

Table 4 compares the maximum adsorption capacity of the CTAB@TBC composite with those of various adsorbents reported in the literature for the adsorption of XO. The results indicate that the CTAB@TBC composite possesses a higher adsorption capacity and its adsorption performance is significantly superior to that of the reference adsorbents, which fully demonstrates the structural merits and application potential of this composite in the field of dye adsorption. This finding not only verifies the capability of CTAB@TBC for the efficient removal of dye pollutants from aqueous solutions but also provides experimental evidence and theoretical support for the development of high-performance adsorbents for practical wastewater treatment applications.

## 3. Materials and Methods

### 3.1. Reagents and Materials

The tomato biomass was sourced from the Shouguang Vegetable Base. CTAB was acquired from Beijing InnoChem Science & Technology Co., Ltd. (Beijing, China). Ethanol of analytical grade was purchased from Laiyang Kangde Chemicals Co., Ltd. (Yantai, China). The reagents referenced above were employed directly without extra pretreatment. XO was produced by Tianjin Kermel Chemical Reagent Co., Ltd. (Tianjin, China).

### 3.2. Preparation of Tomato-Derived Biochar

Tomato straw was initially rinsed with deionized water to remove surface impurities, cut into 2–5 cm segments, and oven-dried at 110 °C for 12 h to reduce moisture content. The dried material was subsequently ground and sieved to obtain 1–2 mm particles, ensuring uniform heating during subsequent processing. The fabricated powder underwent pyrolysis in a tube furnace at 600 °C under argon atmosphere to maintain inert conditions. The temperature was increased from ambient conditions to 600 °C at a rate of 2 °C per minute, followed by maintaining this temperature isothermally for 2 h. After carbonization, the specimens were allowed to cool naturally to ambient temperature. The resulting biochar was further ground, passed through a 200-mesh sieve, and designated as TBC for subsequent applications.

### 3.3. Synthesis of CTAB@TBC

Initially, 5 g of TBC was introduced into 50 mL of CTAB ethanol solution (40 g/L). After ultrasonicating for 15 min, the mixture was stirred magnetically at 300 rpm and 60 °C for 60 min. Subsequently, the reaction mixture was subjected to microwave irradiation at 150 W constant power and 2.5 GHz frequency, with temperature increased to 200 °C and maintained for 10 min to ensure complete interaction between TBC and surfactant. The synthesis was conducted in a 100 mL heat-resistant glass beaker to maintain controlled reaction conditions. After filtration, the solid residue was thoroughly cleansed, dried, and retained for experimental use. The resulting CTAB-modified tomato-derived biochar was designated as CTAB@TBC and employed in follow-up experiments. To verify that the change in adsorption performance was caused by CTAB modification, the TBC samples were also treated in parallel using the same processing procedure as described above.

### 3.4. Characterization

To investigate the physicochemical properties of TBC and CTAB@TBC composites, a series of characterization techniques were employed. The surface morphology and related features were examined using scanning electron microscopy (SEM, JSM-F100, Zeiss, Germany). Structural changes induced by CTAB grafting, as well as information regarding functional groups, were analyzed using Fourier transform infrared spectroscopy (FTIR, WQF-530A, Beifen Ruili, Beijing, China) at room temperature over a scanning range of 400–4000 cm^−1^. The crystal structure and phase composition were analyzed using X-ray diffraction (XRD, Bruker D8 Advance A25, Karlsruhe, Germany) at a scanning rate of 2°/min. The specific surface area was determined using the Brunauer–Emmett–Teller (BET) model based on nitrogen physisorption data collected with an adsorption analyzer (Micromeritics ASAP 2460, Norcross, GA, USA). Thermal stability was evaluated by thermogravimetric analysis (TGA, STA200RV, Hitachi High-Tech, Tokyo, Japan) over a temperature range of 20–800 °C under a nitrogen flow rate of 20.0 mL/min. The Raman spectra of the samples were obtained using a Raman spectrometer (XploRA PLUS, HORIBA, Paris, France) to characterize their molecular structures.

### 3.5. Adsorption Experiments

Adsorption tests were conducted by adding specified amounts of CTAB@TBC (0–1.0 g) to 50 mL XO solutions with initial concentrations in the range of 0 to 200 mg/L. The adsorption process was conducted at controlled temperatures for predetermined durations. The resulting mixtures were filtered through 0.22 μm syringe filters equipped with MCE membranes. UV-Vis spectrophotometry was used to determine the XO concentrations in the filtrates before and after adsorption treatment. Equations (9) and (10) were used to calculate the XO removal efficiency and adsorption capacity of the adsorbent.

(9) $$R=\frac{C_{0}\;-C_{t}}{C_{0}}\;\times \;100\%$$

(10) $$q_{t}=\frac{\;C_{0}-C_{t}}{m}\;\times \;V$$

In these expressions, C_0_ and C_t_ represent the initial concentration and the concentration of XO at time t (mg/L), respectively; V is the volume (L) of XO SO solution; m signifies the mass (g) of CTAB@TBC; and q_t_ denotes the adsorption capacity at time t (mg/g).

## 4. Conclusions

CTAB@TBC composite was successfully synthesized using a simple and environmentally friendly method, allowing for the efficient removal of XO dye from water. The characterization results demonstrate that the surface of the TBC successfully formed a hydrophobic interface after modification with CTAB. This hydrophobic layer not only significantly improved the material’s adsorption affinity for hydrophobic pollutants but also endowed the material with overall hydrophobic properties. The maximum XO uptake was achieved under the following optimized conditions: pH 6.0, temperature of 25 °C, adsorption duration of 40 min, adsorbent dosage of CTAB@TBC of 0.03 g, and an initial XO concentration of 100 mg/L. Under these conditions, a removal efficiency of 90.38% was attained, corresponding to an adsorption capacity of 150.59 mg/g. The impact of anions on XO adsorption was stronger than that of cations, and their inhibitory effects followed the order SO_4_^2−^ > Cl^−^. Kinetic analysis showed that the adsorption process conformed to a pseudo-second-order model, while the equilibrium data were best described by the Langmuir isotherm, indicating monolayer adsorption. In addition, thermodynamic results confirmed that the negative ΔG values reflect the spontaneous nature of the adsorption process. Furthermore, CTAB@TBC maintained its adsorption efficiency over six regeneration cycles, demonstrating its strong potential for effective XO removal. In addition, the adsorption mechanism was elucidated using XPS and FTIR analyses. The adsorption of diphenol orange initially proceeded via physical adsorption, electrostatic attraction, and hydrophobic interactions, and subsequently stabilized through the formation of surface complexes with the active functional groups on the biochar. Overall, these findings confirm that CTAB@TBC is a highly effective adsorbent for the treatment of wastewater containing cationic dyes.

## Figures and Tables

**Table 1:** Kinetic Parameters for XO Adsorption onto CTAB@TBC.

Models	Parameters	Values
Pseudo-first-order	q_e cal_ (mg/g)	145.85
	k_1_ (1/min)	0.5357
	R^2^	0.8344
Pseudo-second-order	k_2_ [g/(mg·min)]	0.0065
	q_e cal_ (mg/g)	152.12
	R^2^	0.9829
Intraparticle diffusion	Phase I	
	k_i_ (mg/g·min^0.5^)	17.6203
	C (mg/g)	80.5294
	R^2^	0.9899
	Phase II	
	k_i_ (mg/g·min^0.5^)	3.7504
	C (mg/g)	126.6515
	R^2^	0.9593
	Phase III	
	k_i_ (mg/g·min^0.5^)	0.1751
	C (mg/g)	149.2616
	R^2^	0.9995

**Table 2:** Isotherm parameters for XO adsorption onto CTAB@TBC composite.

*T* (K)	Langmuir Model			Freundlich Model		
	*q_m_* (mg/g)	*K_L_* (L/mg)	*R^2^*	*K_F_* (mg/g)	*n*	*R^2^*
298	165.30	1.33	0.9257	61.68	4.88	0.7131
318	163.72	1.41	0.9294	60.16	4.82	0.7152
338	145.47	1.50	0.9407	58.17	5.27	0.7073

**Table 3:** Thermodynamic parameters for the adsorption of XO onto CTAB@TBC.

T (K)	LnK_eq_	∆G (KJ·mol^−1^)	∆H (KJ·mol^−1^)	∆S (J/mol·K)
298	13.71	−33.96	2.5001	122.34
318	13.76	−36.39		
338	13.83	−38.86		

**Table 4:** Comparison of the adsorption capacity for XO between CTAB@TBC and the previous literature.

Adsorbent	q_m_ (mg/g)	Reference
Nano magnetite-biosorbent	17.58	[37]
LaZnFe_2_O_4_@NiWO_4_@D400-MMT@CMS/MMA “S_3_”	44.5	[38]
Activated Carbon (AC treated with acid)	138.12	[39]
Activated Carbon (AC treated with base)	112.36	[39]
Bi-porous bioinspired chitosan foams	122	[40]
γ-cyclodextrin-grafted carboxymethyl cellulose	29.9	[41]
CTAB@TBC	150.59	This work

## Data Availability

The original contributions presented in this study are included in the article, further inquiries can be directed to the corresponding authors.
